# Coronary artery surgery outcome differences by sex

**DOI:** 10.1007/s12055-025-01958-z

**Published:** 2025-05-08

**Authors:** Selma Rizvanovic, Melanie Arnreiter, Alissa Florian, Sigrid Sandner

**Affiliations:** 1 Department of Cardiac and Thoracic Aortic Surgery, Medical University Vienna, Spitalgasse 23, 1090 Vienna, Austria; 2 Department of Cardiothoracic Surgery, Weill Cornell Medicine, New York, NY USA

**Keywords:** Coronary artery bypass grafting, Sex differences, Women

## Abstract

Cardiovascular disease remains the leading global cause of death, with coronary artery bypass grafting (CABG) serving as the gold standard for managing complex coronary artery disease. Despite advancements in surgical techniques and perioperative care, women undergoing CABG continue to face poorer outcomes compared to men, including higher operative and long-term mortality, greater rates of graft failure, and an increased incidence of postoperative complications such as stroke and sternal wound infections. Women also exhibit higher readmission rates, worse recovery trajectories, and lower quality of life after surgery. The underlying factors contributing to these disparities are multifactorial. Women typically present with advanced age, a higher burden of cardiovascular risk factors, and smaller coronary artery and graft diameters, all of which pose technical challenges and may reduce graft patency. Additionally, socioeconomic barriers, delayed diagnoses due to atypical symptom presentation, and lower utilization of evidence-based secondary prevention strategies widen the outcome gap. Efforts to address these disparities must include greater representation of women in clinical trials, as well as designing trials dedicated to women, as emphasized by the Randomized Comparison of the Clinical Outcome of Single vs Multiple Arterial Grafts: Women (ROMA: Women) and Revascularization Choices Among Under-Represented Groups Evaluation (RECHARGE) trials. Future strategies should prioritize personalized cardiac care, optimize surgical approaches tailored to sex-specific anatomy, and strengthen secondary prevention adherence in women.

## Introduction

Cardiovascular disease (CVD) continues to be the leading cause of mortality among both women and men globally [1]. Coronary artery bypass grafting (CABG) is the preferred treatment for complex coronary artery disease (CAD) [2] and is one of the most commonly performed cardiac surgical procedures worldwide, with approximately 800,000 CABG procedures performed annually. Despite the growing prevalence of high-risk patients, substantial improvements in CABG outcomes have been achieved during the past years. However, significant sex-based differences in outcomes still persist, with women experiencing poorer outcomes following CABG compared to men, including higher mortality rates and a greater likelihood of postoperative adverse events [3–7]. The underlying mechanisms for different outcomes following CABG between sexes are still not fully understood. Women are more likely to have microvascular dysfunction and coronary microvascular disease (CMD), and to experience ischemia related to concomitant non-obstructive CAD [8]. They present with CAD nearly a decade later than men, and have a higher burden of cardiovascular risk factors at the time of referral for surgery [3, 7, 9, 10]. Moreover, anatomical differences, such as the smaller diameter of coronary arteries [3, 5, 11, 12], along with lower utilization of arterial grafts [10, 13, 14] and a lower rate of complete revascularization [10] impact surgical outcomes. Secondary prevention is less prevalent in women [15–17] despite guideline recommendations [18]. Socioeconomic barriers further exacerbate disparities, particularly among minority groups [19, 20].

Here, we provide a narrative review of the existing evidence on sex differences in outcomes following CABG surgery.

## Surgical outcomes by sex

### Short- term mortality

Higher operative mortality in women undergoing CABG has been consistently reported in literature [6, 7, 9, 13, 21–23]. In a meta-analysis of 112 observational studies including over 5 million patients, 30-day mortality was higher among women compared to men (4.9% vs 3.3%, odds ratio [OR]: 1.40, 95% confidence interval [CI]: 1.35–1.45, *P* < 0.001) [9]. A similar finding was reported in a study based on the Society of Thoracic Surgeons (STS) adult cardiac surgery database which included 1,042,506 patients (25.1% women) undergoing isolated CABG between 2011 and 2018 (OR: 1.26, 95% CI: 1.21–1.30) [23]. Most recently, female sex was identified as a risk factor for increased operative mortality in an analysis from the STS Database involving 1,297,204 patients (24.5% women) who underwent isolated CABG between 2011 and 2020 in the United States (US). The attributable risk for operative mortality associated with female sex was 1.28 in 2011 and 1.41 in 2020, notably without significant improvement over the study period (*P* for trend = 0.38) [6].

### Long-term mortality

Women also face higher long-term mortality rates. In a meta-analysis of 84 observational studies encompassing 224,340 women, a significantly higher incidence of both operative (OR:1.77, 95% CI: 1.64–1.92; *P* < 0.001) and late mortality (incidence rate ratio [IRR]: 1.16, 95% CI: 1.06–1.26, *P* < 0.001) in women was reported [7]. Schmidt et al. analyzed five cohort studies including 102,263 CABG patients (21% women) and found a significantly higher risk of all-cause mortality after 5 years for women (hazard ratio [HR]:1.20, 95% CI: 1.16–1.25) [24]. Further evidence from the meta-analysis of Alam et al. [22] showed higher 1-year mortality (OR: 1.31, 95% CI: 1.18–1.45) and 5-year mortality rates (OR: 1.14, 95% CI: 1.08–1.20) among women. In a study involving 57,943 patients (19% women) who underwent isolated CABG, women had significantly lower survival rates than men at 10 (65% vs. 74%) and 20 years (31% vs. 41%), *P* < 0.001 following surgery [13]. Notably, these differences in outcomes persisted even after adjusting for baseline risk factors [13]. Selected studies examining sex-related disparities in outcomes after CABG are reported in Table 1.

**Table 1:** Selected studies examining sex-related disparities in outcomes after coronary artery bypass grafting

Study	Study type	Number of patients	Outcome women vs. men
Gaudino et al. [6]	Observational	1,297,204 (24.5% women)	Unadjusted operative mortality (2.8% [95% CI, 2.8–2.9] vs 1.7% [95% CI, 1.7–1.7], P <.001)
LaGrange et al. [25]	Meta analysis of observational studies and RCTs	3,971,267 (29.3% women)	Short-term mortality (in hospital or 30-day mortality) (OR 1.32 [95% CI, 1.14–1.52; P < 0.01])
Shi et al. [9]	Meta analysis of observational studies and RCTs	5,003,618 (28.8% women)	30-day mortality (4.9% vs 3.3%; aOR 1.40 [95% CI, 1.35–1.45])
Enumah et al. [23]	Observational	1,042,506 (25.1% women)	30-day mortality (OR 1.68 [95% CI, 1.63–1.73]) (aOR = 1.26, [95% CI 1.21–1.30])
Alam et al. [22]	Meta analysis of observational studies	966,492 (29% women)	30-day mortality (4.2% vs 2.5%, OR 1.66 [95% CI, 1.59–1.74]) 1-year mortality (2.6% vs 1.9%; OR 1.31 [95% CI, 1.18–1.45]) 5-year mortality (9.3% vs 8.2%; OR 1.14 [95% CI, 1.08–1.20])
Schmidt et al. [24]	Meta-analysis of individual patient data (5 cohort studies)	102,263 (21% women)	Mortality in median follow-up of 5 years: (16% vs 13% HR 1.20 [95% CI, 1.16–1.25])
Gaudino et al. [21]	Pooled analysis of Individual patient data (4 RCTs)	13,193 (20,6% women)	MACCE over 5-year of follow up (33.6 vs 28.7, aHR 1.12 [95% CI 1.04–1.21], P = 0.004) Mortality over 5-year of follow up (20.6 vs 17.3, aHR 1.03 [95% CI 0.94–1.14], P = 0.51)
Sandner et al. [26]	Pooled analysis of individual patient data (7 RCTs)	4,413 (17,6% women)	Graft failure (37.3% vs 32.9%, P = 0.02)
Jawitz et al. [10]	Observational	301,309 (25% women)	LITA to LAD revascularization (aOR 0.79 [95% CI 0.75–0.83]; P < 0.001) Complete revascularization (aOR 0.86 [95% CI, 0.83–0.90]; P < 0.001) Multiarterial grafting (aOR 0.78; [95% CI, 0.75 to 0.81]; P < 0.001)
Robinson et al. [7]	Meta-analysis of observational studies	903,346 (24.8% women)	MACE (IRR 1.40, [95% CI: 1.19–1.66]; P < 0.001) MI (IRR: 1.28, [95% CI: 1.13–1.45]; P < 0.001) Stroke (IRR: 1.31, [95% CI: 1.15–1.51]; P > 0.001)

*aHR* adjusted Hazard Ratio, *aOR* adjusted Odds Ratio, *CI* Confidence Interval, *OR* Odds Ratio, *HR* Hazard Ratio, *IRR* Incidence Rate Ratio, *LAD* Left Anterior Descending Artery, *LITA* Left Internal Thoracic Artery, *MACE* Major Adverse Cardiac Events, *MACCE* Major Adverse Cardiac and Cerebrovascular Events, *MI* Myocardial Infarction, *RCT* Randomized Controlled Trial

### Postoperative complications

Women face a significantly higher risk of major adverse cardiac and cerebrovascular events (MACCE) following surgery. In an individual patient data meta-analysis by Gaudino et al. [21] pooling data from four large CABG trials and involving 13,193 CABG patients (2714 women), women were found to have a significantly higher risk of MACCE over a five-year period post-surgery (HR:1.12, 95% CI: 1.04–1.21; *P* = 0.004). Women also had a 30% higher risk of myocardial infarction (MI) (HR: 1.30, 95% CI: 1.11–1.52) and a 22% higher risk of repeat revascularization (HR: 1.22, 95% CI: 1.04–1.43). An important finding of this study was the inverse relationship of the risk of MACCE with age, with outcome disparities between the sexes observed in patients younger than 75 years, but similar outcomes in those older than 75 years. This finding may be explained by the presentation of CAD in younger women. In particular, women are more likely to suffer from CMD, along with coronary spasm and coronary dissection, which may be associated with increased mortality and poorer outcomes post-surgery [21]. Robinson et al. [7] reported in a meta-analysis of 84 observational studies that women had a higher incidence of major adverse cardiac events (MACE) (IRR: 1.40, 95% CI: 1.19–1.66, *P* < 0.001), MI (IRR: 1.28, 95% CI: 1.13–1.45, *P* < 0.001) and stroke (IRR: 1.31, 95% CI: 1.15–1.51, *P* > 0.001). Similar results were reported in a meta-analysis by LaGrange et al. [25] with women showing a higher risk for postoperative stroke (OR:1.31, 95% CI: 1.02–1.67, *P* < 0.01) in propensity-matched studies.

A higher incidence of other postoperative complications among women has also been described [3, 5–7, 12, 21, 27, 28]. In a recent STS Database analysis, women had a higher risk of postoperative complications including stroke, kidney failure, and sternal wound complications [6]. In the setting of bilateral thoracic internal artery (BITA) revascularization, the risk of sternal wound complications tends to be higher in women. In an analysis of over 1.5 million patients who underwent isolated CABG, women who received BITA had a significantly higher risk of deep sternal wound infection compared to those receiving a single internal thoracic artery (OR: 2.99; 95% CI: 2.52–3.55). Moreover, when compared to men, the risk was significantly greater in women (OR: 1.80; 95% CI: 1.56–2.08) [29]. These findings align with those reported in the Arterial Revascularization Trial, in which female sex was identified as a risk factor for sternal wound complications (OR: 1.58, CI 95% 1.07–2.34, *P* = 0.02) [30]. While skeletonization of BITA may lower this risk in men, its benefits in women appear to be more limited [3].

Increased risk of postoperative complications may contribute to higher readmission rates among women. Gupta et al. [5] reported that women had significantly higher adjusted odds of readmission at both 30- and 90-days compared to men (30-day adjusted OR [aOR]: 1.24, 95% CI: 1.21–1.28; 90-day aOR: 1.25, 95% CI: 1.22–1.28). Similar findings were reported in a study of 1113 patients, of which 309 were women, with women having a significantly higher hospital readmission rate then men (20.5% vs. 11%, *P* = 0.005) within the first two months post-surgery [27]. Although data on long-term hospital readmission rates after CABG is limited, a recent post-hoc analysis of the CORONARY trial involving 4623 patients (18,9% women) provided evidence on readmission rates five years after surgery. Time-segmented analyses showed a higher risk for all-cause (HR: 1.21, 95% CI: 1.05–1.40; *P* < 0.001) and cardiac (HR: 1.26, 95% CI: 1.03–1.69; *P* = 0.033) readmission in women after the first 3 years of follow-up. Notably, all-cause readmission strongly correlated with all-cause mortality (Rho coefficient: 0.60, 95% CI: 0.48–0.66), whereas cardiac readmission showed a strong correlation with cardiac mortality (Rho coefficient: 0.60, 95% CI: 0.13–0.86) [28].

### Graft failure

The clinical benefits of CABG surgery are commonly thought to be due to continued graft patency. Graft failure is associated with adverse cardiac events, including myocardial infarction (MI), repeated revascularization, and mortality [31]. The reported incidence of graft failure after 10 years ranges from 10 to 50%, depending on the type of graft used [32], with women showing a higher incidence compared to men [31]. In a pooled analysis of individual patient data from 7 randomized controlled trials (RCTs) involving 4,413 patients, of which 777 (17.6%) were women, graft failure in one or more grafts occurred in 37.3% of women and 32.9% of men one year after surgery (*P* = 0.02). When assessed per graft, graft failure occurred in 20.5% (465 of 2,273) of grafts in women, compared to 15.8% (1,726 of 10,890) of grafts in men (*P* < 0.0001). Higher graft failure rates were observed with saphenous vein grafts (SVG) and right internal thoracic artery (RITA) grafts [26]. The quality of veins in women is generally poorer than in men [26, 33], with factors such as pregnancy, advanced age, obesity, occupations involving prolonged standing, family history and a history of deep venous thrombosis contributing significantly to this disparity [33]. The increased rate of RITA graft failure in women may be attributed to its smaller diameter and greater susceptibility to spasm in women [26]. Smaller diameter vessels may pose technical challenges during surgery, along with reduced short-term patency of conduits grafted to smaller arteries [34]. Additionally, the risk of thrombosis is increased, as smaller arterial lumen is associated with narrower target sites, thereby enhancing the risk of thrombosis [11, 34]. Although the size and flow of the radial artery (RA) are smaller in women as well [35], the use of RA grafts may be particularly beneficial in women [36]. A patient-level meta-analysis of six randomized trials by Gaudino et al.[36] compared RA grafts with SVGs and showed a greater clinical benefit in women regarding MACE (HR: 0.23, 95% CI: 0.09–0.56; P_interaction_ = 0.01). Beyond the smaller diameter of grafts, women’s coronary arteries are also typically narrower and more prone to spasm compared to men’s [3, 11, 12, 21]. Data from autopsy specimens and imaging procedures show that diameters of the mid-left anterior descending artery (LAD) and left main coronary artery in women are 10–15% smaller [37]. In a study evaluating the relationship between postoperative outcome and mid-LAD diameter by O’ Connor et al. [34] it was found that women had a significantly smaller average mid-LAD diameter compared to men (1.81 mm vs. 2.04 mm; *P* < 0.001). Mid-LAD diameter was measured intraoperatively using graduated probes in 0.5-mm increments (range: 1.0–3.5 mm). Smaller target coronary vessel sizes were associated with increased in-hospital mortality rates: 15.8% for vessels measuring 1.0 mm, 4.6% for those measuring 1.5 to 2.0 mm, and 1.5% for vessels measuring 2.5 to 3.5 mm (*P* < 0.001 for trend). Although smaller coronary arteries are more common in women, they can also occur in smaller-stature men, presenting similar procedural challenges. The Coronary artery surgery (CASS) study [38] found that patient size and coronary artery diameter were independent predictors of operative mortality. After adjusting for these variables, sex was not a statistically significant predictor of surgical risk, suggesting that elevated mortality in women may be due to smaller body size and vessel diameter rather than inherent sex-related factors [38]. Furthermore, a study by Limanto et al. [39] analyzed the relationship between target vessel size and postoperative outcomes, showing that larger target vessels were associated with better results, including a significantly reduced risk of graft occlusion (OR: 0.18, 95% CI: 0.05–0.62, *P* = 0.007).

### Postoperative recovery and quality of life

Following CABG surgery, women have a more challenging recovery process than men, with higher levels of depressive symptoms, lower physical functioning (PF), and higher incidence of hospital readmission [27]. In a study evaluating physical and psychological recovery outcomes following CABG, both sexes showed significant improvements in PF and mental health, but the gains were notably greater in men at six months post-surgery [40]. The mean improvements in PF and mental health scores were 14.0 vs. 7.3 (*P* = 0.0002) and 8.9 vs. − 3.0 (*P* = 0.026), respectively. Even after adjusting for baseline characteristics, women had higher rates of adverse outcomes, including hospital readmissions (32.6% vs. 21.2%), worsening functional status (25.7% vs. 11.1%), and mental health decline (17.5% vs. 12.6%) [40]. Challenges that women experience during post-operative recovery may affect their overall quality of life (QOL). In a study conducted by Peric et al. [41], women had worse QOL at baseline as well as post-surgery (across all evaluated sections of the Nottingham Health Profile questionnaire). Although both sexes had improvements in QOL six months after surgery, female sex was identified as an independent predictor of QOL worsening in domain of pain (OR: 3.93, 95% CI: 1.74–8.88; *P* = 0.001). Similar findings were observed in the study by Martin et al.[42], which included 496 patients (17.14% women). Women had a significantly lower health-related QOL at baseline (*P* < 0.01) and six months post-CABG (*P* < 0.01) in all evaluated dimensions. A recent meta-analysis by Masterson et al. [43] evaluated the impact of CABG on QOL, analyzing data from 15 RCTs and over 16,500 patients. The study demonstrated significant improvements in both disease-specific (Seattle Angina Questionnaire) and generic QOL (SF- 36 Physical Component, SF- 36 Mental Component, EQ- 5D) measures following CABG, whereby trials with a higher proportion of men showed better outcomes in generic QOL scores (EQ- 5D).

## Mechanisms behind sex differences

### Differences in risk factors

**Fig. 1:**
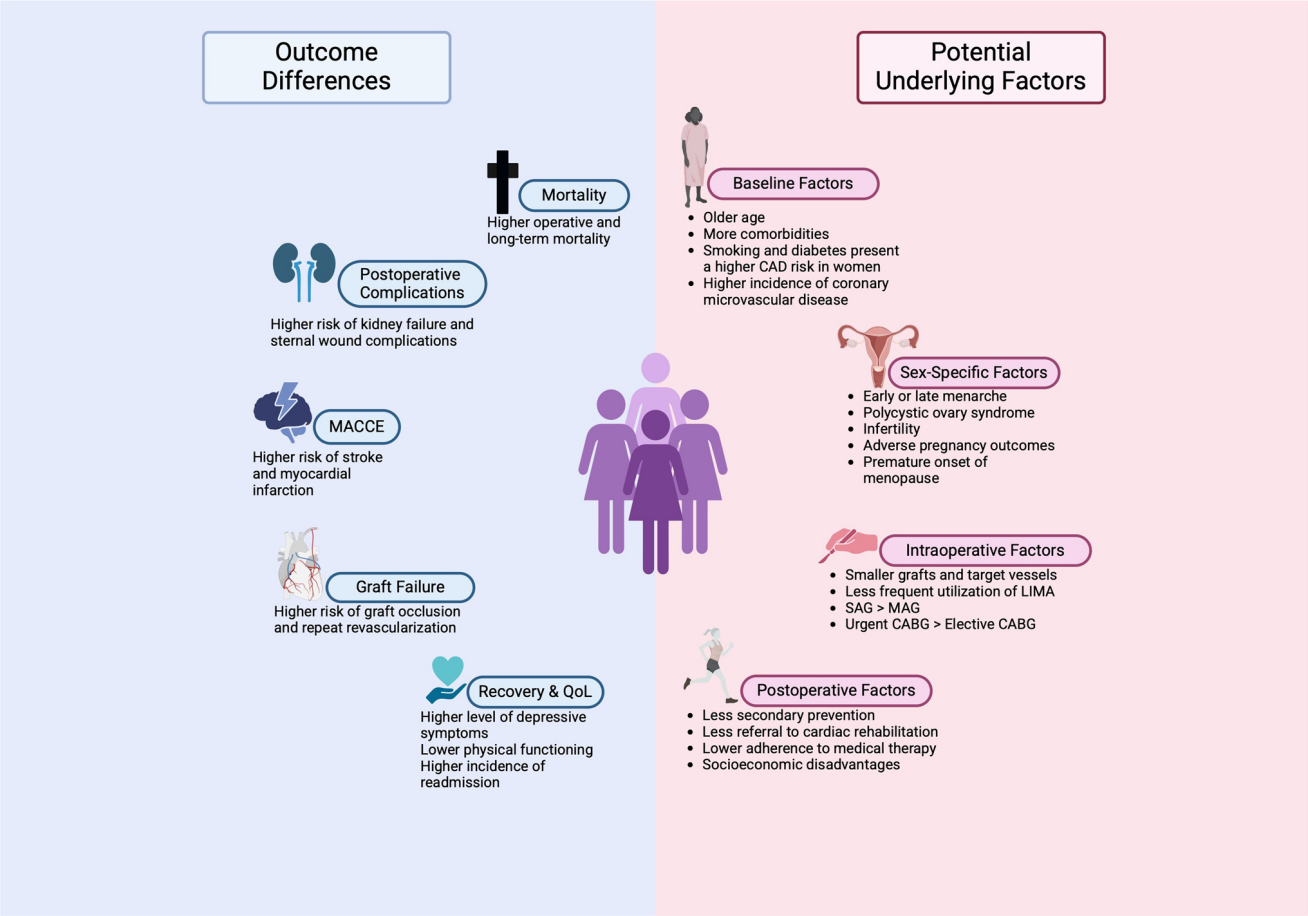
Overview of potential contributing factors to poorer postoperative outcomes in women undergoing coronary artery bypass grafting; CAD: Coronary Artery Disease; CABG: Coronary Artery Bypass Graft; LIMA: Left Internal Mammary Artery; MACE: Major Adverse Cardiac Events; MAG: Multiple Arterial Grafting; QOL: Quality of Life; SAG: Single Arterial Grafting. Created in BioRender. Florian, A. (2025); https://BioRender.com/u04o904

Risk factors for CAD affect men and women differently. Traditional factors such as smoking, hypertension, dyslipidemia, obesity, and diabetes mellitus increase the risk for CAD in women more than in men. Smoking increases the risk for heart disease more significantly in women due to metabolic and hormonal differences [44]. It increases the risk for CAD by 4–6 times in men and 6–9 times in women. Daily smoking increases cardiovascular mortality by 18% in men and 30% in women, which underscores its severe impact on female health [11]. Hypertension, although developing later in women, is generally less well-controlled, thereby increasing their risk. Diabetes is associated with a disproportionately higher risk of CAD in women compared to men [44], and is recognized as an independent risk factor for CAD in women [11]. In addition, even in the pre-diabetic stage, the risk of development of microvascular dysfunction, which may co-exist with obstructive CAD, is significantly elevated in women [11]. Women typically have a more atherogenic lipid profile from infancy to young adulthood and after middle age, but a more favorable lipid profile during the premenopausal years (ages 20–50) [11, 44]. Women typically present with CAD approximately a decade later than men, and have a higher prevalence of cardiovascular risk factors [3, 7, 9, 10]. Certain risk factors and conditions are unique to women [3, 11, 44, 45]. Early menarche is associated with a higher risk of CAD due to its association with obesity and insulin resistance [11, 44, 45]. Approximately 2% to 10% of pregnant women develop hypertensive pregnancy disorders [44] placing them at a significantly higher risk of CVD [45]. Gestational diabetes further increases the risk of CVD later in life [11, 45]. Polycystic ovary syndrome (PCOS) is a common endocrine disorder, with an incidence ranging from 5–13%, which increases the risk of CVD up to 30% [45]. Premature menopause, especially before age 45, is an independent risk factor for CAD, potentially due to a deficiency in protective endogenous hormones, notably estrogen [11, 12, 44]. Figure 1 demonstrates potential contributing factors to poorer postoperative outcomes in women undergoing CABG.

### Symptom presentation

Chest pain is the most common symptom of obstructive CAD, however, women often present with “angina-equivalent” symptoms such as shortness of breath, palpitations, and fatigue, and describe a range of chest pain symptoms [46] that may be initially misdiagnosed as unrelated to CAD. During acute MI, men typically present with severe chest pain, whereas women often present with dyspnea and severe fatigue, with or without chest pain [11]. Dyspnea as a primary symptom of acute coronary syndrome (ACS) occurs in 48% of women compared to 40% of men [47]. Other non-chest pain symptoms include discomfort in the jaw, neck, back, and arms, as well as fatigue, nausea, or symptoms resembling indigestion [47].

Variation in pain patterns, including anginal and non-anginal symptoms or pain, in women may be associated with the increased prevalence of CMD observed in women. Approximately 65% of women presenting with chest pain are found to have CMD [48]. In line with this, women are more likely to experience ischemia related to non-obstructive CAD, while obstructive CAD is more commonly observed in men [8]. Unlike obstructive CAD, CMD cannot be effectively treated with revascularization, which may explain why women often benefit less from the revascularization [6].

### Delay in diagnosis and treatment

Symptom variability often delays diagnosis and treatment, ultimately worsening cardiovascular outcomes [44]. Consequently, diagnostic criteria based on typical CAD symptoms result in approximately 65% of women being misdiagnosed [11]. Additionally, risk assessment scores derived from male population thresholds may inaccurately estimate cardiac risk in women [3]. Despite these limitations, such scores remain widely used as standardized risk assessment tools in clinical practice [49], further highlighting the need to refine risk assessment methods by including sex-specific factors for greater accuracy.

### Disparities in surgical approaches

Sex differences also extend to surgical approaches, particularly in graft selection for CABG. Multiple studies have reported that women typically receive fewer grafts on average, with less frequent utilization of the left internal thoracic artery (LITA) and multiple arterial grafts (MAG), when compared to men [10, 13, 14]. In a study of 10,915 patients who underwent CABG with at least one arterial graft, of whom 2,961 received MAG, the MAG utilization rate was significantly higher in men compared to women (34% vs 28%, *P* < 0.001) [14]. Similar results were reported by Attia et al. [13] in a study of 57,943 CABG patients, with women receiving fewer arterial grafts, including BITA grafts. Moreover, Jawitz et al. [10] showed that LITA (aOR: 0.79; 95% CI: 0.75–0.83; *P* < 0.001) and MAG utilization rate (aOR: 0.78; 95% CI 0.75–0.81; *P* < 0.001) were lower in women. In addition, data from a study of 12,736 CABG patients, including 3,163 women, showed that women received fewer distal arterial anastomoses than men (no distal arterial anastomosis: 3.4% vs 1.8%, *P* < 0.0001; 1 distal arterial anastomosis: 89% vs 84% *P* < 0.0001; 2 distal arterial anastomoses: 6.5% vs 12%, *P* < 0.0001). [12]. Similar findings were reported by Gaudino et al. [50] in a study from the New York State Cardiac Surgery database which included 63,402 patients (24% women), showing that 21.9% of men received 2 or more arterial conduits, compared to 13.8% of women, confirming the persistence of this gap across multiple studies. The reasons for the lower rate of MAG in women are not yet fully understood. The smaller size of both the grafts and the target coronary arteries in women may present greater technical challenges and potentially influence the lower adoption of MAG in women compared to men [3]. Furthermore, the limited availability of randomized data supporting the clinical benefits of MAG in women may also contribute to its underuse in this patient population [51].

The use of coronary endarterectomy (CE) provides additional level of complexity. Notably, study data reveal that CE is more frequently performed in men [52, 53].

In a recent analysis of the STS database, women were shown to have a lower median nadir intraoperative hematocrit (22.0%, Q1-Q3:20.0%− 25.0% vs 27.0%, Q1-Q3:24.0%− 30.0%; standardized mean difference 97.0%) than men, with a stronger association between operative mortality and nadir intraoperative hematocrit at hematocrit values < 22.0% (*P* < 0.001) [54].

### Secondary prevention after surgery

Sex disparities exist in both primary [15] and secondary prevention [16, 17] and in adherence to cardiovascular medications [55, 56]. Antiplatelet and lipid-lowering medications are essential for secondary prevention post-CABG. Aspirin reduces graft occlusion, lowers adverse cardiovascular event risk, and improves long-term survival, while statins decelerate atherosclerosis progression and reduce adverse events [18, 57]. Despite the efficacy of statins [18, 58, 59] and aspirin [18, 60, 61] for both sexes and guideline recommendations [18], women are less likely to receive these therapies [15–17]. Statin and aspirin prescriptions remain underutilized in high-risk groups, with women still receiving significantly lower secondary prevention efforts [62]. Additionally, therapy adherence is lower among women [55, 56], potentially due to a higher incidence of adverse drug reactions (ADRs). This may be associated with sex-specific pharmacodynamic and pharmacokinetic differences making women more prone to adverse drug reactions, including variations in drug absorption, metabolism, and clearance, often leading to higher systemic drug exposure. Additionally, sex-specific factors such as hormonal influences, immune response variations, and differences in body composition contribute to altered drug effects and increased susceptibility to adverse events. Moreover, polypharmacy, including over-the-counter and herbal supplements, is more prevalent in women, further increasing the risk for potential drug interactions and ADRs in women [56].

However, a recent nationwide study involving 15,448 patients (18.7% women) who underwent CABG over a nine-year period reported promising developments in statin use for secondary prevention among women. In this study women had the same likelihood as men to receive statin prescriptions over five years of follow-up after CABG (aOR: 1.03, 95% CI: 0.92–1.16). Notably, women were more likely than men to fill prescriptions for high-intensity statins during the same period (aOR: 1.12, 95% CI: 1.02–1.23) suggesting a significant improvement in the use of statins [63]. Additionally, high-intensity statins were associated with a greater reduction in the risk of death (HR: 0.52, 95% CI: 0.42–0.63, *P* < 0.0001) compared to low/moderate-intensity statins (HR: 0.61, 95% CI: 0.50–0.75, *P* < 0.0001), with similar effects observed in both men and women (p_int_ = 0.477). An increased focus on secondary prevention, may help reduce the disparities observed in outcomes between the sexes.

### Differences in recovery

Social factors, particularly traditional gender roles, may have a significant impact. Women often experience greater disruption in their lives due to traditional responsibilities related to home management and caregiving. These challenges are particularly pronounced among younger women, where family and caregiving demands are typically greater. Women often attempt to resume household responsibilities early, despite facing significant recovery challenges, further complicating their postoperative recovery period [27].

## Strategies to address disparities

Women are underrepresented in cardiovascular trials [4]. Given the disparities observed in cardiovascular outcomes between men and women, it is important that future studies prioritize sex-based research, as emphasized by the National Institutes of Health [64] a few years ago. Despite this, many studies continue to fail to appropriately report sex-specific data, contributing to an ongoing research gap. Increasing awareness among researchers and clinicians about the significance of sex differences in cardiovascular health is crucial to close this gap and promote more informed clinical decision-making.

While significant progress has been made in cardiac surgery, much remains to be done. There is an urgent need for more RCTs that focus specifically on women. In this context, the ongoing ROMA:Women trial [51] (ClinicalTrials.gov: NCT04124120), which focuses on multiple arterial grafting in women, aims to fill this gap by generating prospective data that will guide the development of optimal grafting strategies. Similarly, the ongoing RECHARGE trial [65], which compares revascularization modalities (CABG vs. percutaneous coronary intervention) among under-represented populations, women (ClinicalTrails.gov: NCT06399692) and minority groups like Black and Hispanic patients (ClinicalTrials.gov: NCT06399705), aims to address the existing disparities and contribute to the development of evidence-based data tailored to these patient groups.

## Conclusion

Although CABG is a firmly established surgical procedure, women experience poorer outcomes following CABG, including both short- and long-term outcomes. To bridge this gap and ensure that women benefit equally from surgical revascularization, there is an urgent need for more inclusive research, updates to clinical guidelines, and tailored treatment strategies. Future studies must focus on personalized cardiac care that accounts for sex-specific factors, ensuring that both men and women receive the most effective evidence-based treatment.

## Data Availability

Not applicable. No new data were generated or analyzed in this review.
